# Qualitative investigation of the experiences of older people living with persistent pain and frailty and their decision to seek support: findings from the POPPY-Q study

**DOI:** 10.1136/bmjopen-2025-104744

**Published:** 2025-10-27

**Authors:** Nicola Harrison, Rahena Mossabir, Anne Forster, Nicky Kime, Amanda C de C Williams, Lesley Brown

**Affiliations:** 1 Academic Unit for Ageing and Stroke Research, Bradford Institute for Health Research, Bradford, UK; 2 Bradford Institute for Health Research, Bradford, UK; 3 Psychology & Language Sciences, UCL, London, UK

**Keywords:** Frailty, Chronic Pain, QUALITATIVE RESEARCH, Aging

## Abstract

**Abstract:**

**Objectives:**

Persistent pain is common among older people living with frailty and can impact on their daily living, mobility, social interactions and sleep. However, healthcare support to mitigate impact is lacking in this population. The Pain in Older People with Frailty (POPPY) study is a multiphase, mixed-methods study that addresses how pain management services for older people with frailty should be organised and delivered.

**Design:**

For this phase (POPPY-Q), we used qualitative methods: semi-structured interviews with a grounded theory approach to analysis.

**Setting and participants:**

Community-dwelling older people (≥75 years) with persistent pain and frailty were invited to participate in two qualitative interviews (in-person/remotely) 10 weeks apart. Interviews took place in varied geographical locations across England between July 2022 and August 2023 and explored experiences of living with pain and access to and engagement with services and healthcare professionals (HCPs) and support and treatments received for pain.

**Results:**

Twenty-six people (77–91 years) with pain and frailty (from mild to severe) consented and were interviewed; 24 completed a second interview. Three interviews included a spouse/family member. Themes were general health and well-being; pain and its impact; acceptance of living with pain; support-seeking decisions; experience of accessing support; and perception/experience of pain support and treatment. This paper focuses on *pain acceptance* and *support-seeking; other themes are used contextually, and accessing support was rare*. Many participants were stoical about pain; some prioritised other health conditions; some preferred self-management; some were resigned and had lost hope of effective treatment; some expressed concern about burdening healthcare resources.

**Conclusions:**

HCPs should be aware of the stances of older people with frailty about seeking support for pain and should be proactive, asking about pain. Longer appointments for complex cases may allow general practitioners to address pain, offer reassurance, provide information or referral or arrange a follow-up consultation focused on pain management.

**STRENGTHS AND LIMITATIONS OF THIS STUDY**

- The Pain in Older People with Fraity (POPPY-Q) study included a well-characterised group of older people living with frailty, from varied living circumstances, geographic locations and socioeconomic backgrounds.
- Most participants had mild or moderate frailty; few with severe frailty participated due to health issues.
- This study did not include older people who lacked capacity; this population requires dedicated attention but was beyond the scope of this study.
- This study did not include individuals living in care homes, who often have dependency on others for essential activities of daily living and limited mobility and as such require dedicated research.

## Introduction

 Chronic pain, persistent pain of at least 3 months duration, is common among older people.^1^ Prevalence ranges from 25% to 76%, depending on the population sampled and methodology used.^2 3^ Pain can be classified as chronic primary pain, pain that cannot be explained by another condition or where the pain itself is the main problem, rather than a symptom of something else, or as chronic secondary pain, which is pain that results from an underlying condition.^4^ People may experience both chronic primary and secondary pain at the same time.^4^

Frailty is a disorder of several inter-related physiological systems that results in a depletion in homeostatic reserve and vulnerability to disproportionate changes in health status after relatively minor events. As a result, individuals become more vulnerable to significant health deterioration following relatively minor stressors, such as a change in medication or a routine surgical procedure.^5^ Frailty affects approximately 12% of people aged 65 years and over, and approximately one-third of people over 80 years.^6^ Frailty is considered a long-term condition requiring long-term strategies and interventions.^7 8^ This has been facilitated by the ability of UK general practices to readily identify older people living with frailty for appropriate services using the electronic Frailty Index (eFI).^9 10^

Pain prevalence of between 31% and 60% was reported in a review of cohort and cross-sectional studies of community-dwelling older people with frailty.^11^ Older people with frailty are approximately three times more likely to experience intrusive pain compared with older people without frailty.^12^ Additionally, pain impacts on their mobility, ability to accomplish tasks, socialise and to sleep.^12^ Pain is associated with an increased risk of incident frailty and worsening frailty,^13 14^ and an association with increased mortality and dementia in older people.^15^

Persistent pain is experienced within a social context, and variations in personal relationships, community engagement and healthcare settings can influence how older people adapt to and manage pain.^16^ Social isolation can impact on pain and disability in older people.^16^ Given that older people often face substantial social losses, such as bereavement, social status and reduced independence, along with difficulties in maintaining social connections, these factors can compound the experience of persistent pain.^16 17^ Additionally, persistent pain may lead to greater social withdrawal, as older people reduce their participation in social and recreational activities.^16 18 19^

The high prevalence of pain and its impact among older people living with frailty highlights a significant unmet need. This may arise from factors including personal decisions not to seek treatment or support, choices not to initiate treatment or limited access to appropriate services tailored to this population’s needs. Managing pain in older people with frailty is particularly challenging due to the frequent presence of multimorbidity and the complexities associated with polypharmacy.^20^ It is important to understand the experiences of older people living with both frailty and persistent pain, as well as the barriers and enablers that influence their support-seeking decisions. Pain, and particularly its impact, is potentially modifiable and should be recognised as a priority for this underserved group.

The POPPY study is a mixed-methods, multiphase, co-design study that aims to develop the evidence and associated service models to support older people living with frailty to manage their pain and to reduce its impact on their lives.^21^ This paper focuses on findings from qualitative interviews with older people undertaken within the larger work programme. The study protocol^21^ and systematic review^22^ are reported elsewhere. Findings from interviews with healthcare professionals will be reported separately. We refer to this component of the study as the POPPY-Q study, to distinguish it from the overall programme.

### Aims and objectives

The overall study aim is to develop the content, delivery mode, implementation strategies, service and professional support and guidance to enable older people with frailty to better manage their pain and reduce its negative impact on their lives, relationships, functioning and quality of life.

The objective of the POPPY-Q study was to undertake qualitative interviews with older people living with frailty and persistent pain in order to explore how they experience pain, their interactions with services and healthcare professionals, the support and treatments they received and to understand what service models are required to optimise access and provide support, as part of the larger work programme.

## Methods

Methods for the overall programme are reported elsewhere.^21^ The methods employed for the POPPY-Q study are detailed below.

### Ethics

The study protocol was approved by Leeds-East Research Ethics Committee on 28 April 2022 (22/YH/0080).

### Study design

Qualitative methods were employed using semi-structured interviews. A constructivist grounded theory approach was used^23^ incorporating simultaneous data collection and analysis and constant comparison. This inductive and reflexive methodology is appropriate for developing explanatory theories about processes underlying a particular phenomenon. The experiences of older people with frailty in living with and seeking support for persistent pain have seldom been explored. As such, a *bottom-up* approach helped to understand the decisions, actions and meanings that shape their experiences.

### Patient and public involvement

Patient and public involvement (PPI) contributors with lived experience, either personally or as carers, were actively engaged from the study outset to ensure the research was relevant and accessible to the needs of the population being studied. Their involvement in the POPPY-Q study included reviewing and refining participant-facing materials; participating as ‘mock’ interviewees to inform the development of topic guides; co-creating case study vignettes; contributing to the interpretation of findings; and reviewing lay summaries of findings for dissemination (see Gripp2 short form in online supplemental file 1).

### Identification of potential POPPY-Q participants from the Community Ageing Research 75+ (CARE75+) study

**Figure 1:**
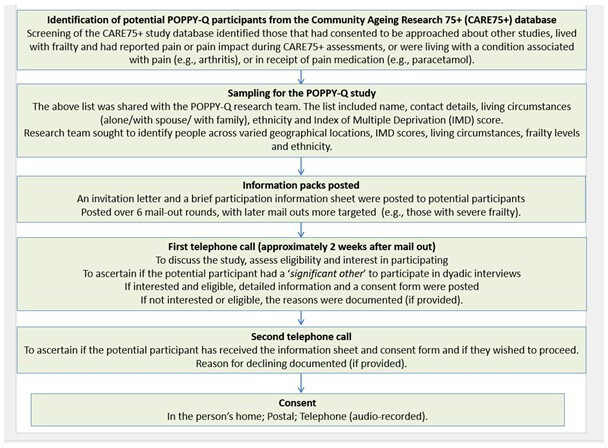
POPPY-Q study identification and recruitment. POPPY, Pain in Older People with Frailty

POPPY-Q participants needed to be community-dwelling, ≥75 years and living with persistent pain and frailty.^21^ They were current or past participants from the longitudinal cohort Community Ageing Research 75+ (CARE75+) study (ISRCTN16588124)^24 25^ (figure 1). The CARE75+ study recruits older people aged ≥75 years and collects observational health, social and economic outcome data. The CARE75+ study has broad inclusion criteria and only those living in care homes and towards the end of life (estimated life expectancy of ≤3 months; receiving palliative care) are ineligible. CARE75+ assessments are conducted in person^24^ or remotely^25^ at 6 monthly or yearly intervals for participants’ duration in the CARE75+ study. Information on medication, comorbidities and eFI score^9^ is derived from their General Practice Electronic Health Record. A Fried frailty score^26^ is limited to those receiving in-person assessments only.^24^

To be eligible for POPPY-Q, participants required a Fried frailty^26^ score of between 1 and 5 or an eFI score ≥0.13.^9^ Frailty status was based on their latest CARE75+assessment data.

### Sampling for POPPY-Q

The CARE75+ project manager screened the CARE75+ database^24 25^ to identify those who had agreed to be contacted about other studies; were living with frailty;^9 26^ had reported pain or pain impact during their CARE75+ research assessments; were in receipt of medication prescribed for pain (e.g., paracetamol); or had a condition associated with pain (eg, osteoarthritis).

The above data represented the most recent information available about participants from the CARE75+ dataset and varied for each potential participant. CARE75+ assessments were conducted between 3 months and 2 years prior to the list being shared with the POPPY-Q team. In some cases, participants had already completed the CARE75+ study but had provided ongoing consent to be approached about other studies.

This list was shared with the POPPY-Q research team. The list included names and contact details, living circumstances, ethnicity and Index of Multiple Deprivation scores^27^ (but not pain duration). From this list, the research team purposively sampled from varied geographical locations and levels of deprivation,^27^ living situations, ethnic backgrounds and levels of frailty to ensure a diverse sample and capture a broad range of views (this represents a minor deviation from the protocol^21^). Invitations were posted in batches over six mail-out rounds. This approach allowed the team to reflect on who had consented and adjust subsequent invitations accordingly. For example, in later rounds, individuals with moderate to severe frailty^9^ and those who identified with an ethnicity other than White-Caucasian were specifically invited, as these groups were underrepresented in earlier invitation rounds.

### Recruitment to POPPY-Q

We aimed to recruit up to 30 older people, including some with a spouse/partner or family member and conducting two interviews per participant. Following discussions within the Programme Management Group (PMG), this number was considered sufficient to capture the views of the target population in varied living circumstances, from a mixture of locations, spanning the least deprived and most deprived locations, to meet the requirements of the overall work programme,^21^ and accounting for participants taking part in two interviews.

Potential participants were screened for persistent pain^28^ and eligibility during the initial telephone contact by the researchers, NH or RM. Those deemed eligible and interested in finding out more about the study were posted a detailed participant information sheet and followed up by a telephone call by NH or RM to establish interest and answer any questions. Fully informed consent was provided by participants in writing or verbally (see Participant Consent Form in online supplemental file 4).

### Data collection

Two in-depth, semistructured interviews were conducted predominantly in the older person’s home, with the option of telephone or remote interviews (via Teams) if preferred.

First interviews were conducted by NH or RM and facilitated participants’ reflection on their pain experience, the impact pain has on their lives, their access to and experience of services, healthcare professionals and support and treatments received for pain. Between interviews (approximately 10 weeks), participants were encouraged to document instances where pain influenced their decision-making and actions, using a method of their choice (eg, written notes). Recognising the characteristics of the study population, documentation was optional. The second interview focused on participants’ reflections on how they managed living with pain during the intervening 10 weeks. This was followed by a discussion around potential improvements to pain services for older people living with frailty.

Topic guides were informed from guidelines,^29^ study PMG members and the PPI oversight group.^21^ Questions were piloted with PPI members to ensure they prompted meaningful discussion and were developed as an iterative process and revised after initial interviews to reflect emerging topics (see topic guides for interviews 1 and 2 in onlinesupplemental files 3^5^).

### Data analysis

All interviews were audio-recorded with participants’ informed consent and transcribed verbatim. Transcripts were read multiple times to ensure thorough familiarisation with the data. Data collection and analysis were conducted concurrently, consistent with grounded theory methodology. Researchers NH and RM employed an inductive, line-by-line, open coding approach, focusing specifically on identifying actions and processes in the initial set of transcripts. Coding was conducted independently by NH and RM and then reviewed collaboratively to generate initial categories through axial coding. Subsequent transcripts were divided between NH and RM and coded according to emerging categories, through axial coding and employing constant comparison throughout. This process led to the development of a preliminary conceptual model.

Subsequent meetings between NH and RM facilitated discussion and refinement of categories and subcategories, enhancing the emerging model’s clarity and coherence. Any discrepancies were resolved through consensus. Ongoing meetings were also held with co-author and co-applicant, AW, to discuss established categories and to ultimately validate the conceptual model. The finalised coding framework was then applied to the complete dataset, including follow-up interview transcripts, using a theoretical coding approach that aligned with the established categories. Coding and data management were supported by NVivo software V.12.^29^ To ensure participant confidentiality, pseudonyms were assigned to all individuals.

### Reflexivity

None of the participants were known to the researchers personally. NH recognised that being both a carer for a family member living with persistent pain and having previously worked as a social worker in adult social care may have influenced both the data collection and analysis processes. This personal and professional connection to the research topic may have shaped her interpretations, particularly through the lens of her caregiving experience. To address this, NH maintained reflexive memos throughout the study, regularly documenting thoughts, emotional responses and underlying assumptions as they emerged during interviews and data analysis.

The phenomenon of living with pain in old age, particularly in the context of long-term conditions, is something RM had been familiar with through past research and from supporting her mother who has lived with arthritis for over 20 years. RM previously viewed pain as a symptom of other conditions, in which case focusing on pain as the primary topic of investigation in this present study required a perceptual shift. Many of the stories shared by the participants resonated with RM’s mother’s experience of managing her arthritis and interactions she has had with healthcare professionals, which may have led RM to uncritically conduct some parts of the data collection process and make certain assumptions about the data during the analysis process. These potential biases were addressed through regular discussions about the data with colleagues and the involvement of PPI in the interpretation of data. Further, while the study sample comprised mainly White-Caucasian older people, as someone of South Asian ethnicity, RM felt she was able to build a stronger rapport with the two participants who were of Pakistani origin. Although RM did not speak the same language as these two participants, and most of the interview was conducted in English, they shared aspects of their family life and daily routine with the understanding that RM was familiar with their cultural elements and did not require further explanation.

Employed by the National Health Service (NHS), NH and RM recognised the potential impact this could have on participants’ willingness to openly share their views and experiences of healthcare services, in which case they often explained to participants their role as a researcher and reassured them that the information they provided would be kept confidential.

While there was an age gap between NH and RM and the participants, engaging in ‘small talk’ before the interview enabled NH and RM to further relay that they were interested in their stories told in their words. This informal approach also to some extent mitigated the impact of researcher-participant power imbalance.

To improve rigour and trustworthiness, reflexivity was embedded throughout the research process. NH and RM engaged in ongoing critical reflection by maintaining detailed reflexive memos to document potential biases and assumptions alongside regular analytical discussions with colleagues. We did not check the content of the transcripts with POPPY-Q participants as we considered this task to be too burdensome for an older frail population, who had already undertaken two interviews for this study. There was, however, substantial PPI involvement in the analytical process which provided an external perspective, helping to validate the findings with older people. These strategies collectively enhanced the credibility, dependability, confirmability and transferability of the study.^30^

## Results

### Findings

**Figure 2:**
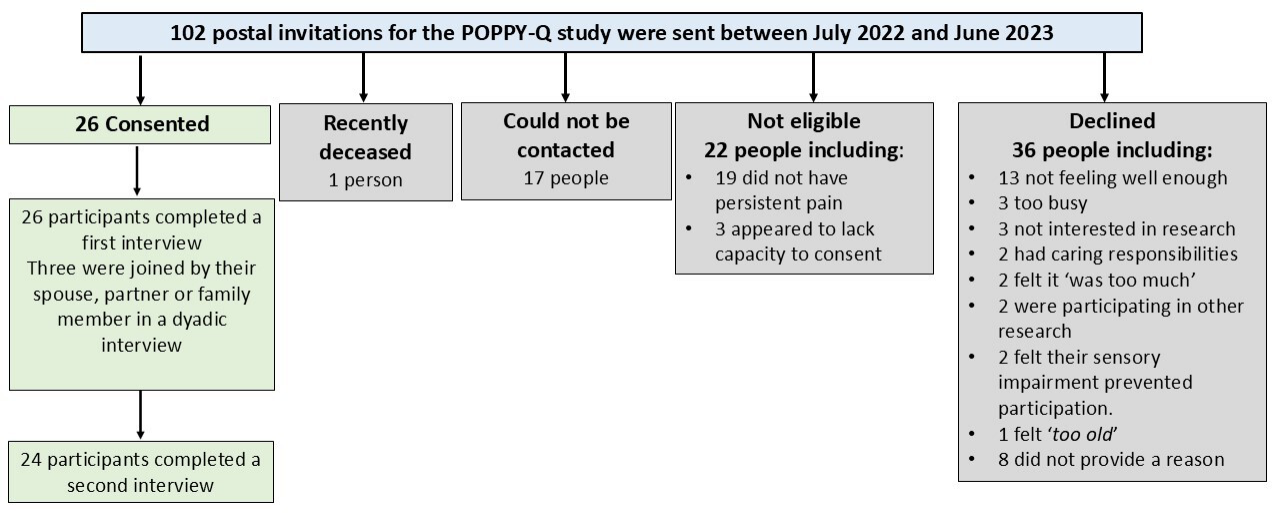
POPPY-Q consented participants and reasons for not participating. POPPY, Pain in Older People with Frailty.

Invitations were posted to 102 older people (figure 2).

Twenty-six participants completed a first interview and twenty-four completed a second interview. Interviews lasted between 35 and 103 min. Three participants documented their pain experience in a written format between interviews and noted instances where pain influenced their decision-making and actions. Three participants undertook a dyadic interview with their significant other. Participants lived with persistent pain and frailty caused by various health conditions, some with pain in multiple sites and caused by more than one health condition. Seven participants reported experiencing recent pain (within the last 5 years) and the remainder reported long-standing pain (≥5 years duration). See table 1 for characteristics of the included participants.

**Table 1:** Characteristics of included participants in the POPPY-Q study (n=26) (recruitment: July 2022–August 2023)

Pseudonym	Age (at time of first interview)	Sex	Self-reported ethnicity	Living circumstances	Frailty score *eFI	Frailty score Fried†	Number of reported pain site(s)	Number of comorbidities (general practice electronic health record)
Jack	Early 80s	Male	White	Alone	0.28		3^+^	6
Donald	Early 80s	Male	White	Partner/spouse	0.25		2	2
Derek	Early 80s	Male	White	Alone		1	1	0
Barry	Early 80s	Male	White	Alone		1	2	2
Francis	Late 80s	Female	White	Alone	0.25		3	3
Elizabeth	Early 80s	Female	White	Partner/spouse		1	3^+^	2
Arthur	Late 80s	Male	White	Alone	0.25		3^+^	6
Bernard	Late 80s	Male	White	Partner/spouse		3	1	4
Brianˆ¶	Early 80s	Male	White	Partner/spouse		1	3^+^	4
Veronica	Early 80s	Female	White	Partner/spouse		1	2	5
Susan	Mid-80s	Female	White	With family		1	1	3
Christopher¶ˆ	Early 80s	Male	White	Partner/spouse		1	3^+^	3
Janet	Early 80s	Female	White	Alone	0.22		2	4
Terence	Early 80s	Male	White	Alone		1	1	4
Helen	Late 80s	Female	White	Alone		1	1	12
Mavis‡	Late 80 s	Female	White	Partner/spouse		1	2	6
Marjorie	Early 80s	Female	White	Partner/spouse	0.39		3^+^	9
James‡	Early 90s	Male	White	With family		1	1	8
Stuart	Late 70s	Male	White	Partner/spouse	0.25		3^+^	5
§Ahmed	Early 80s	Male	South Asian	Partner/spouse	0.33		3^+^	Not available
§Ruqayah	Early 80s	Female	South Asian	Partner/spouse	0.31		3^+^	3
Beryl‡	Mid 80s	Female	White	Alone	0.56		3^+^	13
Vera	Early 80s	Female	Black Caribbean	Alone	0.31		3^+^	Not available
Frank	Early 80s	Male	White	Alone	0.31		2	10
Mary	Early 80s	Female	White	Alone	0.28		3^+^	7
Yvonne	Mid 80s	Female	White	Alone	0.25		2	4

* eFI scores: fit (a score below 0.12); mildly frail (0.12 to 0.24); moderately frail (0.24 to 0.36); severely frail (0.36 and above).

† Fried scores: not frail (0); pre-frail (1-3); frail (3-5).

‡ Dyadic interview included the participant with pain and their significant other.

§ Both participants had persistent pain—interviewed together (married couple).

¶ Did not take part in a second interview

eFI, electronic Frailty Index.

Frailty was not a term that participants used to describe themselves. However, participants reported weakness, fatigue, slowing down and tasks taking longer. Many participants had employed someone to garden or clean or had family support for household tasks (eg, shopping). Other common problems reported included falls, incontinence, poor balance, cognitive decline and poor grip strength.

Participants were distributed across deprivation deciles, with 12.0% living in the most deprived areas (first–second deciles). A further 44.0% were in the third–fifth deciles, and 40.0% were in the sixth–eighth deciles. Only 4.0% of participants lived in the least deprived areas (ninth–10th deciles). The 10th decile represents the 10% of areas with the lowest levels of deprivation (least deprived).^27^

**Figure 3:**
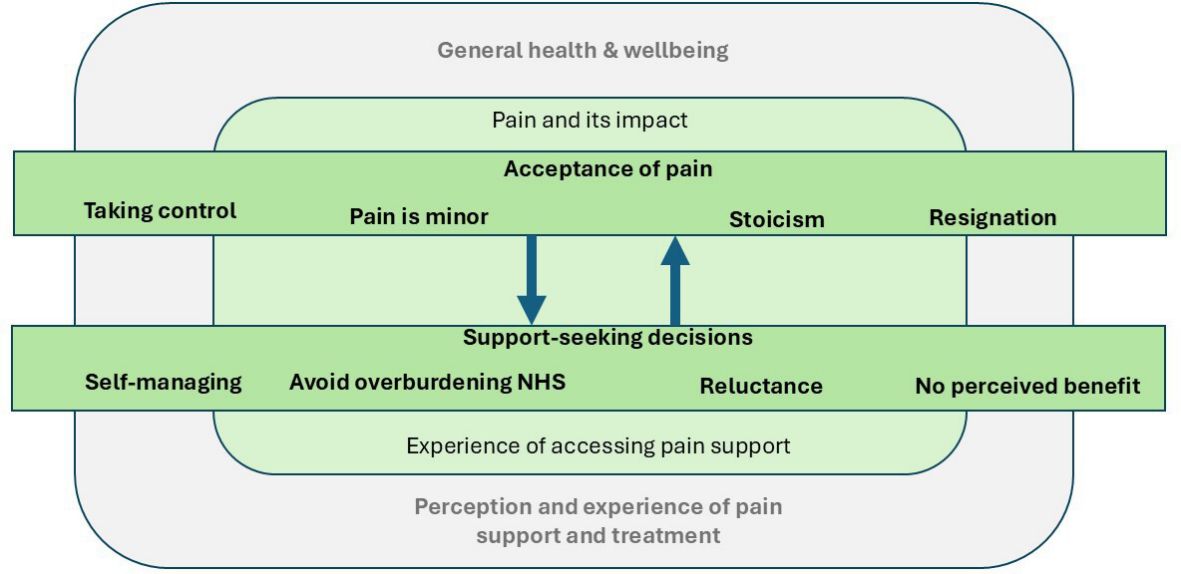
Conceptual model of categories. NHS, National Health Service.

Six categories emerged from the interviews, forming a conceptual model which presents an understanding of how older people experience and respond to persistent pain. The categories are: general health and well-being; pain and its impact; acceptance of living with pain; support-seeking decisions; experience of accessing pain support; and perception and experience of pain support and treatment (figure 3).

No new categories emerged from the dyadic interviews. The two outer categories (general health and well-being and perception and experience of pain support and treatment) provide useful background information and important *context* as to *how* and *why* older people have come to accept living with pain and make decisions around support and treatment. Data such as weight, mood, fatigue and comorbidities were categorised under general health and wellbeing. Perception and experience of pain support and treatment considered, for example, pharmacological and non-pharmacological interventions, highlighting both positive and negative experiences. The two inner categories (pain and its impact and experience of accessing pain support) considered participants’ pain history, severity, frequency and the impact pain has on their daily lives, social life and relationships, for instance and experiences of accessing pain support included barriers to accessing primary and secondary care. Data in this category showed there was limited experience and knowledge of pain services or pain management programmes among those interviewed. These two inner categories are more directly tied to participants’ daily challenges of living with pain and their experiences of accessing support and have a stronger influence on attitudes towards pain and their decisions to seek support for pain.

In the foreground of the model are *acceptance of living with pain* and *support-seeking decisions*. These are dynamically interlinked as participants’ beliefs, approaches and attitudes to living with pain (pain acceptance) can influence and inform whether or not they seek support for their pain (support-seeking decisions). Equally, decisions about seeking support for pain may reinforce current attitudes and beliefs held about living with pain. These categories are novel in research in this population and furthermore are accessible to intervention, so can help inform how services should be organised and delivered for older people living with persistent pain and frailty, as part of the overall work programme (21) (see list of all categories and subcategories in online supplemental file 2).

### Acceptance of living with pain

This category considered the range of attitudes and approaches expressed by participants about living with persistent pain. We have termed this as *Acceptance of living with pain,* and it is comprised of four subcategories: feeling in control; perceiving pain to be minor compared with other health conditions or the experiences of others*;* stoically dealing with pain; and resignation about living with pain. See table 2 for *Acceptance of living with pain* categories.

**Table 2:** *Acceptance of living with pain*: findings from the POPPY-Q study (July 2022–August 2023)

Category	Subcategory	Representative quotes
**Acceptance of living with pain**	*Feeling in control* Having a proactive, determined attitude to living with pain	‘…My theory is it’s not going to rule me. I’m in charge whether I put up with it or not’ (Helen) ‘I do my best, every single day, trying to make sure I do nothing which will obviously aggravate it enough to do that…you’re mindful of what you’re doing, looking where you’re going, being careful’ (Stuart)
	*Perceiving living with pain to be minor compared with other health conditions or the experiences of others* Using (social) comparison to downplay and minimise pain	‘…heart, lungs and liver, I’ve got angina as well, and I’ve got my chest things, I’ve got primary biliary cirrhosis of the liver, I’ve just been diagnosed with macular dystrophy…I had a stroke, in that eye…the other pains I’ve got, I mean I just get on with it really’ (Marjorie) ‘Well, you know if you look around there’s somebody always much worse than what you are so you know they need their attention more than I do’ (Beryl)
	*Stoically dealing with pain* Tolerating, enduring and suppressing thoughts and feelings related to pain	‘I don’t moan about it, I don’t complain, I just get on with life, you know, that’s me…’ (Brian) ‘…I tend to ignore it, I suppose, tend to accept that it’s par for the course and you've got to get on with it’ (James) ‘No good dwelling on things because if you do you could go into depression badly, couldn’t you?’ (Marjorie) ‘I basically think my generation have been used to putting up with things, you know?’ (Yvonne)
	*Resignation about living with pain* Normalising pain and expressing a loss of hope	‘I’ve sort of given up hope that it’s ever going to get better…’ (Stuart) ‘I think anybody at my age will have something and it’s like an old car…and bit by bit, you know, the body'll be going’ (Derek)

POPPY, Pain in Older People with Frailty.

### Feeling in control

Some participants talked about wanting to be in control and maintain their quality of life despite living with pain. They took a proactive approach to living with pain and used words such as ‘fight’ and ‘in charge’ to describe their attitude and determination to not let pain dominate or impact too much on their lives.

Some talked about how they had become attuned to their body and over time had developed better awareness of their physical limitations (because of frailty and pain) and being careful and cautious were ways of maintaining control of their life and pain.

### Perceiving living with pain to be minor compared with other health conditions or the experiences of others

Several participants felt fortunate that they only experienced *minor* pain rather than (as they perceived) more *serious* pain. Some participants compared their pain with other peoples’ health conditions which were perceived to be more significant, such as cancer. They believed others were *worse off* and in more need of support or treatment and consequently often deemed themselves fortunate.

A few reported more worrisome or significant health problems taking precedence in their life and over their pain, such as diabetes and liver cirrhosis. They felt that the impact, burden and attention needed for such conditions were greater than the burden and impact pain has on their lives, and as a result tended to downplay their pain.

### Stoically dealing with pain

Many participants were stoically dealing with pain and considered themselves, or aspired to be, the type of person who tolerates pain without complaining. They tried to make the best of their situation by staying positive and learning to live with pain.

Ignoring and not dwelling on the pain was an important coping strategy to deal with pain and associated worries and anxieties. Some participants found that redirecting their thoughts and attention away from their pain could provide relief.

Some participants considered this stoical approach, in the face of pain and other life adversity, and having lived through the Second World War and its after-effects, to be a generational attitude.

### Resignation about living with pain

Some participants expressed a resignation about living with pain as they believed nothing further could be done. The length of time participants had experienced persistent pain varied, and some with long-term pain expressed little hope of any change in their situation.

Many normalised their pain and indicators of frailty, as they considered it an inevitable part of the ageing process. They felt there was little that could be done to prevent pain from occurring and were resigned to living with pain and other conditions they associated with old age.

We found that *pain acceptance* encompassed a range of attitudes and approaches which are not discrete, and participants expressed these simultaneously. For example, Susan thought her pain was minor, felt fortunate that there was nothing more (serious) wrong with her, and felt pain was an inevitable part of ageing.

*Well, like I say I just thank goodness that’s all I’ve got wrong with me because at my age you know, so I think I’m one of the lucky ones… it’s just a pain, that old age pain.*

### Support-seeking decisions

*Support-seeking decisions* encompass how individuals’ beliefs, behaviours and experiences influence their choices and actions in seeking help for persistent pain. We identified four subcategories: self-management strategies for controlling pain and pain impact; wanting to avoid overburdening the health system and barriers to access for support and treatment; reluctance to seek professional support and treatment for as long as pain is tolerable; and believing that further professional support and treatment will not be beneficial. See table 3 for *Support-seeking decisions* categories.

**Table 3:** *Support-seeking decisions*: findings from the POPPY-Q study (July 2022–August 2023)

Category	Subcategory	Representative quotes
**Support-seeking decisions**	*Self-management strategies for controlling pain and pain impact* Using strategies like pacing and keeping busy to control pain and pain impact	‘Walking helps, yeah, so I go exercise, I walk around, sometimes when the weather is nice, I go twice a day…’ (Vera) ‘If I mow the lawn out there, I get back ache and I have to come in and sit down for a bit…have a cup of tea and we’re back to normal…’ (Barry) ‘I’m not a person who would go to the doctor normally. As you probably know, older people tend not to go to the doctor. But I tend to say, “Well, I’m going to try and see if I can sort this out myself”’ (Derek)
	*Wanting to avoid overburdening the health system and barriers to access for support and treatment* Having concerns about scarce healthcare resources and past negative experiences of access to healthcare	‘…because of the present state of the world and the NHS and I don’t want to bother them with things like this and I can cope with it’ (Donald) ‘…if medical care was more easily accessible you might be more inclined to try…whereas the present situation is it’s hard enough to get an appointment for something serious and so you tend to say…I don’t want to bother them with this, this is trivial…’ (James) ‘I'd got so many different ailments that when I do go to the doctor I can only really bring up one because they haven't got the time for more, and then it’s deciding what’s the most urgent one’ (Mary) ‘Oh, I give up, I give up trying to see a doctor a couple of years ago…life’s too short to spend…45 min in a morning wanting somebody to answer the damn phone’ (Frank) ‘But telephone conversation, I’m sorry, you need to see somebody and get what they’re like, yeah, you do, and that all seems to be, face-to-face seems to be finished. And plus the fact you never see the same doctor’ (Christopher)
	*Reluctance to seek professional support and treatment for as long as pain is tolerable* Self-evaluating pain and need for professional support	‘I think it’s gentle enough for me to forget about it but that’s all I can say. Ordinarily, I might have considered going to the doctor to say what was happening and possibly to have another injection, but I shan’t, I’ll tolerate it’ (Bernard) ‘Well, if I suddenly felt I couldn’t walk or the pain came back. Yes. I mean the excruciating pain’ (Janet) ‘…I had bad hips for about two or 3 weeks, thought well I can’t do this, so I went and seen him…’ (Brian)
	*Believing that further professional support and treatment will not be beneficial* Feeling disillusioned with treatment options or efficacy	‘No, there’s no point in going on about pain to the GP because they either, you know, say take paracetamol, or no, or they refer you for physio’ (Francis) ‘…we've tried out different drugs and they've…gave me hallucinations and upset the kidneys, so we've had to stop them. …she (GP) put me on another one…and this caused problems and it made the pain worse’ (Mary) ‘Well we’ve just got to live with it, doesn’t matter how many tablets you take or what like that, the pain’s still there’ (Arthur)

GP, general practitioner; NHS, National Health Service; POPPY, Pain in Older People with Frailty.

### Self-management strategies for controlling pain and pain impact

Some participants shared self-management or coping strategies developed to try and stay in control of their pain and minimise their pain and pain impact. For many, these strategies had evolved over many years and were used if support and treatment had been deemed inadequate to manage their pain; employed in conjunction with support and treatment; or in some cases used as an as an alternative to seeking or receiving professional support and treatment.

Many talked about the benefits of staying as busy and active as possible, as participating in activities can distract from, and ease the pain. These activities were often retained from their life before pain. Although fatigue, slow walking speed, weakness and pain can impact on the ability to undertake activities to the same degree as they previously had, one participant still danced a little at home, two rode exercise bikes, some swam and many walked, to distract and relieve their pain.

Many managed pain impact and ensured they did not exacerbate their pain by pacing their activities and including rest breaks into their routine. Others knew that over-exertion one day would lead them to needing to rest the following day.

Some participants had found their own ways of self-managing their pain and frailty by adapting and managing activities of daily living by using aids and equipment to support them, such as sitting to prepare meals or to pray, or using a walking stick when out shopping.

For a minority, self-management was considered the only option as they had not or were unwilling to seek support or treatment. For others, it had been a long time since they had sought any support, but most participants had consulted a general practitioner (GP) about their pain at some point in their lives. However, many reported a lack of discussion about pain management in primary care.

Some participants sought information and advice for their pain from other sources, to support self-management. For example, one participant had searched on the internet about his ongoing chest pain following heart surgery and had self-diagnosed his condition, and another participant had looked on the internet as she had not received any information following her diagnosis of facet joint syndrome. Some participants had received advice or had learnt from friends’ and family members’ experience about pain and pain management to support their own self-management.

### Wanting to avoid overburdening the health system and barriers to access for support and treatment

Some participants considered their pain not serious enough to warrant the use of what they considered to be overstretched NHS resources. Some reported not wanting to waste their GP’s time as they considered their pain to be minor. For some, current difficulties within the NHS prevented them from seeking support. As such, they self-rationed their contact with healthcare services.

Many participants lived with multiple health conditions, such as chronic obstructive pulmonary disease, diabetes and kidney disease, and the majority were on long-term medication and had care plans in place for these conditions. Seeking support for these conditions, rather than their pain, was often their priority.

The limitations of only being able to discuss one health problem per appointment with a GP were expressed by several participants, and having to decide on the most important health concern during a consultation appeared to be a barrier to reporting pain.

Lengthy telephone queues to contact the GP surgery were an issue raised by many and were considered burdensome and frustrating, discouraging participants’ from seeking support. If participants did access their GP surgery, many were dissatisfied with the lack of continuity of care and having to speak to a different GP each time. The lack of face-to-face appointments since the COVID-19 pandemic was also an issue raised among participants. These concerns created further barriers to reporting pain.

### Reluctance to seek professional support for as long as pain is tolerable

Some participants were reluctant to seek support for their pain if it was ‘tolerable’ but would seek support if their pain became ‘severe’ enough or started to impact daily living and functioning, such as the ability to undertake personal care tasks. These participants would conduct a self-evaluation of pain symptoms and the impact of their pain on their daily lives and decide *if* and *when* professional support was required.

### Believing that further professional support and treatment will not be beneficial

Some participants reported that the treatment options available for their pain were unsatisfactory, particularly pharmacological interventions. These options were not always aligned with what participants wanted or expected, and this mismatch served as a barrier to accessing further support.

Pharmacological treatments, such as paracetamol and codeine, were commonly offered, but many participants expressed concerns about side effects (eg, constipation and dizziness), leading some to discontinue their use. Others were worried about the risk of addiction and reported taking medication only when deemed absolutely necessary or stopping altogether. Additionally, some participants chose not to take pain medication due to the inconvenience and burden of managing multiple treatments simultaneously. Others reported being unable to use such medications due to specific contraindications. Some were taking regular pain medication, although a number reported that it had little effect on their pain. Several participants had tried various treatments for pain over the years and had lost hope of finding an effective solution.

For some, their condition had been described by their GP as age-related ‘wear and tear’ that did not require further investigation. While some participants accepted this explanation, others were dissatisfied with the response and the absence of further support, feeling dismissed due to their age. This experience created a barrier to further reporting their pain.

## Discussion

This paper contributes to the existing literature on the attitudes and decision-making processes of community-dwelling older people living with persistent pain, with relevance to those experiencing frailty and multiple health conditions. We identified a range of perspectives and approaches related to pain acceptance, shaped by contextual and experiential factors and varied support-seeking behaviours. Some participants were satisfied with their approach to managing pain. Over time, they had developed bodily awareness, an understanding of their physical limitations associated with frailty and pain and recognition of factors that exacerbated pain. Subsequently, some felt confident in their ability to self-manage their pain, using strategies such as physical activity, pacing and incorporating rest when needed. Preferred coping strategies were often self-directed and familiar.^31^

However, many participants adopted a stoical attitude, placing value on not complaining and seeking to distract themselves or minimise the significance of their symptoms. Such behaviour is consistent with previous research and is recognised as a potential barrier to the expression and reporting of pain among older individuals.^32^ It has been reported that a stoical/reticent approach to living with pain and subsequent under-reporting is more commonly observed in older populations compared with their younger counterparts.^33^

The POPPY-Q study identified a sense of resignation among some participants, marked by a diminished sense of hope and a perceived lack of control over their condition. Underpinning this resignation appeared to be the belief that pain is an inevitable consequence of ageing. This outlook seemed to be shaped by individuals’ own perceptions of ageing and, at times, by messages conveyed by healthcare professionals, similar to other findings.^34^ While pain may be an inevitable consequence of certain age-related conditions, its impact can often be mitigated with appropriate support, such as psychosocial approaches to pain management. However, older individuals who view pain as an unavoidable aspect of ageing may be less inclined to seek such support.^35^

Most participants were living with at least one other long-term health condition, and many appeared to downplay their experience of pain in relation to what they perceived as more serious conditions, such as diabetes. This tendency is consistent with previous findings^36^ on the impact of persistent pain in older people. This research also found unique differences in perceptions and attitudes of older people regarding their pain, in comparison to the ill-health of their peers (social comparison). Social comparison, defined as evaluating one’s own characteristics or behaviours relative to others,^37^ emerged as a strategy used by some participants to downplay their pain. They compared themselves to others they perceived as being *worse off*. This may contribute to older people’s reluctance to seek help or treatment and can be detrimental, as it may prevent individuals from accessing the support they need.^38^

This study identified a tendency among participants to under-report pain to healthcare professionals, or at least a lack of recent consultation about their pain. While some participants felt they were successfully managing their pain and did not seek additional support, others demonstrated stoicism, resignation or a focus on managing other health conditions. Some were reluctant to seek help while their pain remained tolerable, and others had lost confidence in the availability of effective treatment, sometimes due to previous experiences of rejecting treatments or being told that no further options were available. These behaviours were influenced by concerns about being a burden on the NHS, heightened awareness of resource constraints and negative experiences with accessing telephone or face-to-face services. Collectively, these factors appeared to contribute to self-rationing, echoing findings from previous research.^38^

Within this study, older people with frailty were often living with multiple health conditions and polypharmacy, and some had clear care plans in place to manage these. However, there was limited evidence of clear plans for pain management, even though many were well accustomed to following guidance and instructions for other health conditions.

The majority of participants had no prior knowledge or experience of pain services, resulting in a lack of findings regarding existing service models for pain management. However, this lack of knowledge, experience or reported opportunity to access such services was itself an important finding and highlights the need for more targeted support for older, frailer adults, ensuring they are provided with opportunities to develop effective pain management skills.

### Strengths and limitations

The study sample comprised a well-characterised group of older people living with frailty, representing a variety of living circumstances, geographical locations and socioeconomic backgrounds. The majority of participants had mild to moderate frailty, with comparatively limited representation from individuals with more severe frailty. Frailty scores were derived from the most recently available CARE75+ dataset. However, for some former CARE75+ participants who subsequently took part in POPPY-Q, these scores may have been recorded up to 24 months prior to their participation in the current study. Although individuals with severe frailty were invited to participate, many declined due to health-related issues.

This study did not include individuals lacking capacity, a group that warrants dedicated attention but was beyond the study’s scope. This study did not include older people living in care homes. While pain management is a significant concern for this population, care home residents often experience high rates of dementia, dependence on others for essential daily activities and limited mobility,^39^ highlighting the need for dedicated research into effective pain support strategies for this population.

Interviews and analysis were undertaken by researchers without a clinical background. NH had a background of working with older people in a social work capacity, and RH within a research capacity. There will be some researcher influence on the interpretation of the findings in light of their previous experience of older people.

### Practice and policy implications

Older people with frailty are frequent users of health and social care services,^40^ providing multiple opportunities for healthcare professionals to enquire about their pain. This population requires reassurance that reporting pain to GPs or other healthcare providers is appropriate and that doing so does not place an undue burden on services. This population often has multiple comorbidities and is affected by polypharmacy, making the provision of an effective conversation about pain management challenging within a standard 10-min consultation. Some general practices have adopted 15-min appointments, reflecting British Medical Association recommendations^41^ for extended consultations in complex cases. Longer appointments may allow GPs to proactively address pain, offer reassurance, provide appropriate information or referrals, or arrange a dedicated follow-up consultation focused on pain management.

General practices and other healthcare services should ensure the availability of accessible, evidence-based resources about pain management, such as educational materials and links to web-based guidance. However, healthcare professionals must consider the prevalence of digital exclusion among older, frailer populations,^42^ recognising that online resources may not be accessible for all.

Referral to specialist pain services may benefit certain individuals, especially where community-based services are available. Pain services typically deliver psychologically informed approaches to help manage persistent pain, and older people with frailty should have access to such support to develop pain management skills. However, further research is needed to identify which individuals would benefit most from a referral and to optimise referral timing.

A 2021 report of Core Standards for Pain Management Services (CSPMS) UK recommended the establishment of early biopsychosocial assessment within community settings to ensure principles of self-management are available early to service users with persistent pain conditions^43^ and integrating with public health services in prevention and early intervention at community level of care to reduce or prevent persistent pain-related disability. The CSPMS proposes that where possible, care pathways should be developed and used by multidisciplinary teams and informed by user groups to support effective pain management provision within local communities.^43^ The integrated care for older people implementation framework provides guidance for systems and services to support health and social care systems to respond to the complex needs of older people.^44^ This may provide a tool to consider services through a structured, system-wide approach.^44^

The Comprehensive Geriatric Assessment is a multidisciplinary process to evaluate the medical, psychological and functional status of older people, to inform treatment.^45^ Pain assessment is not a formal component; however, its inclusion might help identify and address pain in this population, supporting a more holistic approach. This seems particularly relevant considering older people’s reluctance towards seeking support for their pain.

Pain, and its impact, is potentially modifiable. Older people with frailty should be equipped with the knowledge, support and confidence to manage it. Failing to do so risks leaving this population to suffer avoidable distress, reduced quality of life, and unnecessary loss of independence.

## Conclusions

Healthcare professionals should be aware of the beliefs and attitudes of older people with frailty and persistent pain, as well as the barriers they face in reporting pain and accessing support. Proactive enquiry about pain is an important first step in addressing the needs of this underserved group. To enable this, decision-makers should review care pathways to ensure that older people have adequate opportunities to report pain during routine health and social care interactions and that relevant healthcare professionals have sufficient time to ask about pain with scope for a productive discussion and consideration of next steps. Extending consultation times during general practice interactions could provide an opportunity to address pain for this population, who often have complex needs.

## Supplementary material

10.1136/bmjopen-2025-104744 online supplemental file 1

10.1136/bmjopen-2025-104744 online supplemental file 2

10.1136/bmjopen-2025-104744 online supplemental file 3

10.1136/bmjopen-2025-104744 online supplemental file 4

10.1136/bmjopen-2025-104744 online supplemental file 5

## Data Availability

Data are available upon reasonable request.
