# Supplementary material for: Qualitative investigation of the experiences of older people living with persistent pain and frailty and their decision to seek support: findings from the POPPY-Q study
Source: BMJ Open. 2025 Oct 27;15(10):e104744. doi: 10.1136/bmjopen-2025-104744 (PMC12570947; doi:10.1136/bmjopen-2025-104744)
Supplement: online supplemental file 1 [file bmjopen-15-10-s001.pdf]

GRIPP2 short form

Section and topic	Item	Reported on page No
1: Aim	Report the aim of PPI in the study	5
2: Methods	Provide a clear description of the methods used for PPI in the study	5 & 18
3: Study results	Outcomes—Report the results of PPI in the study, including both positive and negative outcomes	5, 9, 10
4: Discussion and conclusions	Outcomes—Comment on the extent to which PPI influenced the study overall. Describe positive and negative effects	9, 10
5: Reflections/critical perspective	Comment critically on the study, reflecting on the things that went well and those that did not, so others can learn from this experience	Not covered

PPI=patient and public involvement
