# Supplementary material for: Qualitative investigation of the experiences of older people living with persistent pain and frailty and their decision to seek support: findings from the POPPY-Q study
Source: BMJ Open. 2025 Oct 27;15(10):e104744. doi: 10.1136/bmjopen-2025-104744 (PMC12570947; doi:10.1136/bmjopen-2025-104744)
Supplement: online supplemental file 2 [file bmjopen-15-10-s002.pdf]

THE POPPY-Q

Categories/sub-categories

1.General health and wellbeing

- 1.1 Comorbidities/multi-morbidities
- 1.2 Weight management
- 1.3 Tiredness
- 1.4 Weakness
- 1.5 Staying active/staying healthy

2. Pain condition and its impact

- 2.1 Pain condition(s) and history
  - *2.1.1 Duration of pain*
  - *2.1.2 Progression of pain*
  - *2.1.3 History of pain treatments*
- 2.2 Pain impact
  - *2.2.1 Severity/intensity*
  - *2.2.2 Frequency and triggers*
  - *2.2.3 Activities of Daily Living*
  - *2.2.4 Sleep*
  - *2.2.5 Mental health/wellbeing*
  - *2.2.6 Social Activities*
  - *2.2.7 Physical Symptoms*
  - *2.2.8 Caring Responsibilities*
  - *2.2.9 Impact on relationships*
  - *2.2.10 Adaptation of ADL*

3. Acceptance of living with pain

- 3.1 Feeling in control
- 3.2 Perceiving pain to be minor to other health conditions or the experiences of others
- 3.3 Stoically dealing with pain
- 3.4 Resignation about living with pain

4. Support-seeking decisions

- 4.1 Self-management strategies for controlling pain and pain impact
- 4.2 Wanting to avoid overburdening the health system and access issues for support and treatment

4.3 Reluctance to seek professional support for as long as pain is tolerable
4.4 Believing further professional support and treatment will not be beneficial

5. Experience of access to accessing pain support

4.1 General healthcare experience
4.2 Presentation in primary care
4.3 Referral to specialist service
4.4 Other options/private treatment

5. Perceptions and experience of different pain support and treatments

5.1 Pharmacological treatments
5.2 Non-pharmacological treatments
5.3 Surgery
