# Supplementary material for: Qualitative investigation of the experiences of older people living with persistent pain and frailty and their decision to seek support: findings from the POPPY-Q study
Source: BMJ Open. 2025 Oct 27;15(10):e104744. doi: 10.1136/bmjopen-2025-104744 (PMC12570947; doi:10.1136/bmjopen-2025-104744)
Supplement: online supplemental file 3 [file bmjopen-15-10-s003.pdf]

Appendix 1.

Topic Guide for POPPY-Q study qualitative interviews with older people (first interview). V3.0

TOPIC GUIDE

Interview 1: Older people

1. Experience of living with pain

1.1 Can you tell me a bit about your pain?

- What type(s) of pain do you have? Do you know the cause of your pain?
- How long have you had the pain(s)?
- How would you describe the pain(s)? How does it/they feel?
- If you have a number of pains, which pain(s) is most problematic? **Shall we focus on this/these pain(s) throughout the interview?**
- How frequent is the pain(s)? Do you experience any variation in your pain? Is it episodic or there all the time?
- What things might make your pain worse?
- What do you do to relieve the symptoms? How effective are these?

1.2 Can you tell me if and how your pain affects your daily life?

- Does it affect your self-care like washing and dressing?
- Does it affect your ability to cook and clean?
- Does it impact on your mobility?
- Does it affect your ability to get out and about like doing shopping and seeing friends/family?
- Does it affect your mood and how you feel?
- Does it impact on sleep?

1.3 Can you think of ways you might have changed things in your life because of your pain?

- To continue doing things that you like to do such as hobbies and activities.
- Do you have any aids/adaptations to help you manage your daily life due to pain?

1.4 Do you receive any support from a spouse/family member(s) or friend(s) due to living with pain? If so, with what areas of daily living and how much support do they provide?

To establish if there is support from a spouse/ family member/friend prior to asking this question.

- Has living with pain affected your family life or important relationships, if so how?
- How do you feel about the support you receive from family/significant other(s)

2. Access to and experience of pain treatments/services

2.1. Who have you consulted in the past about your pain? (GP, Consultant, Physio, other HCP). **Breakdown each interaction in turn.**

If so, how easy or difficult was it to make contact with them?

- If so, how did you feel about bringing this subject up?
- If so, did you feel listened to and understood?
- If so, did you find this/these conversations helpful?
- What were your expectations from this conversation? Were they met?
- If not, has anything prevented you from making contact with HCP's?

2.2 Did you receive an assessment and diagnosis into your pain? What treatment(s) have you been offered for your pain, if any?

- How did you feel about the assessment you received?
- How did you feel about the diagnosis you received?
- How did you feel about the treatments you received or were referred for? Did you feel these treatments were appropriate for you and met your needs at the time?
- If you received any of the treatments, how effective or appropriate were they in treating the pain?

2.3 Have you sought or been given advise about your pain and how to manage it?

- Peers/family
- HCP's
- Own research

2.4 Were you asked about what would help you manage your pain by HCP's?

2.5 Have you had a review of your pain medication and pain management following any treatment by HCP's?

3. Additional comments

3.1 Is there anything else about how you manage your pain that you would like to share with me?

3.2 Did you feel happy with the topics we discussed?
