# Supplementary material for: Qualitative investigation of the experiences of older people living with persistent pain and frailty and their decision to seek support: findings from the POPPY-Q study
Source: BMJ Open. 2025 Oct 27;15(10):e104744. doi: 10.1136/bmjopen-2025-104744 (PMC12570947; doi:10.1136/bmjopen-2025-104744)
Supplement: online supplemental file 4 [file bmjopen-15-10-s004.pdf]

ID □□□□□□□□

The POPPY Study
Participant Consent Form

	Items 1-10 are mandatory<br>I confirm that…	Initials in the box
1	I have read the Participant Information Sheet for the POPPY study (V 1.1) and the study has been explained to me. I have been given the opportunity to ask questions and am satisfied with the answers provided.	
2	I understand the reason for the research and what will happen if I take part.	
3	I understand that my participation is voluntary and that I am free to withdraw at any time, without giving any reason.	
4	I agree to take part in two interviews, up to 10 weeks apart. Interviews will be conducted in person, by telephone or by video call.	
5	I agree to my voice being recorded during the interview.	
6	I understand that the information I give will only be used for the purposes of research, and that personal details will be treated with the strictest confidence.	
7	I understand that any data or information used in any publications, workshops or presentations which result from this study will be anonymous, so no-one will be able to identify me personally in any way.	
8	I agree to the use of anonymised direct quotes in publications, presentations and other outputs from the POPPY study.	
9	I agree to my details, including my name and address, and a copy of this consent form being stored at Bradford Royal Infirmary for the purpose of managing this study.	
10	If I withdraw from the study (or am withdrawn from the study), non-identifiable information already collected will be retained and used in the study unless I specifically request otherwise.	
**Items 11 to 13 are optional**		
11	I agree to the researcher accessing information I previously provided to the CARE75+ study.	
12	I agree to be **invited** to co-design workshops at a later date to help inform pain service and I am aware that I can decline this invitation.	
13	I agree to my spouse/partner, relative or an unpaid carer to be invited to take part in the interviews with me (if applicable).	

ID □□□□□□□□

Participant consent	
Participant’s name	
Participant’s signature	
Date	
Researcher’s name	
Researcher’s signature	
Date	
Date audio consent recorded (if applicable)	

**I confirm that I want the following designated contact person to be asked about my wishes to continue in the study if I become unwell during the course of the study (optional).**	
Name	
Telephone number	
Email	
Adress	
