# Supplementary material for: Qualitative investigation of the experiences of older people living with persistent pain and frailty and their decision to seek support: findings from the POPPY-Q study
Source: BMJ Open. 2025 Oct 27;15(10):e104744. doi: 10.1136/bmjopen-2025-104744 (PMC12570947; doi:10.1136/bmjopen-2025-104744)
Supplement: online supplemental file 5 [file bmjopen-15-10-s005.pdf]

TOPIC GUIDE

Interview 2: Older people

1. Reflecting on past 10 weeks of pain and pain management.

To discuss with participants the information they have recorded over the 10 week period since their first interview. The following questions will be used as a guide for prompting participants to talk about some key areas of focus during this discussion:

1.1. In the last 10 weeks, have you experienced any changes in your pain and the impact that it has had?

- Have changes in your pain been better, worse or varied?
- Thinking about things you might have recorded in your diary, what things have helped you to manage your pain and what things have not?
- For example, were there things were you able to do and things you found more difficult?

1.2. In the last 10 weeks, have you needed any more or less support than usual because of your pain?

- What kind of support have you required/ not required?
- Were you able to get the required support at the right time?
- If so, who provided this support?

1.3. In the last 10 weeks, have you had any contact with healthcare professionals regarding your pain?

- Who have you seen/ spoken to about your pain?
- What was the outcome?
- Did you find this helpful?

2. Improving pain services and pain management programmes for older people with frailty

To ask participants to think about their experience of pain and the support and treatments they have received (have a summary of this information from interview 1 at hand).

2.1. Do you think access to healthcare professionals and/ or pain services could be improved to better meet your needs? What type of changes would you suggest?

2.2. Do you think assessment of pain could be improved to better meet your needs? What type of changes would you suggest?

2.3. Do you think communication and information provided by healthcare professionals about pain could be improved to better meet your needs? What changes would you suggest?

2.4. Can you think of ways in which pain treatments/ pain services (if any) that you have experience of can be improved to better meet your needs? What changes would you suggest?

2.5. Do you think the involvement of family members by services delivering treatment for pain can be improved to better meet your needs? What change would you suggest?

2.6. Thinking about the changes you have suggested for improving pain services:

- What positive impact do you think it will have on your daily life?
- What positive impact do you think it will have on your spouse/ family member who supports you?

3. Additional comments

3.1 Did you feel happy with the topics we discussed?
