# Supplementary material for: Qualitative investigation of the experiences of older people living with persistent pain and frailty and their decision to seek support: findings from the POPPY-Q study
Source: BMJ Open. 2025 Oct 27;15(10):e104744. doi: 10.1136/bmjopen-2025-104744 (PMC12570947; doi:10.1136/bmjopen-2025-104744)
Supplement: Supplementary file 6 [file bmjopen-2025-104744.reviewer_comments.pdf]

PEER REVIEW HISTORY

BMJ Open publishes all reviews undertaken for accepted manuscripts. Reviewers are asked to complete a checklist review form and are provided with free text boxes to elaborate on their assessment. These free text comments are reproduced below.

ARTICLE DETAILS

Title (Provisional)

A qualitative investigation of the experiences of older people living with persistent pain and frailty, and their decision to seek support: findings from the POPPY-Q study

Authors

Harrison , Nicola; Mossabir , Rahena; Forster, Anne; Kime, Nicky; Williams, Amanda C de C; Brown, Lesley

---

VERSION 1 - REVIEW

**Reviewer**	**1**
**Name**	**Weber, Orest**
**Affiliation**	**Lausanne University Hospital, Psychiatric Liaison Service**
**Date**	**03-Jul-2025**
**COI**	**None**

General Assessment:

This is a well-written and conceptually sound manuscript that makes a valuable contribution to the literature on older adults living with pain. While similar studies have explored older people's experiences of pain and communication with healthcare providers, this paper's specific focus on frailty and its contextualization within the structure of the UK National Health Service (NHS) offers new and relevant insights. These findings significantly enhance our understanding of how care organization and social ideologies intersect with personal experience and professional interactions.

However, a few clarifications and refinements are needed before publication.

____

Major Points for Revision

1. Abstract – Balance Between Methods and Results

The abstract currently devotes too much space to methodological details and too little to the study's key findings. This imbalance detracts from the paper's potential impact, especially for

readers scanning abstracts to gauge relevance. Please revise the abstract to better reflect the central findings and their implications.

2. Clarify Sampling Strategy (POPPY-Q and CARE75+)

There appears to be some inconsistency in the description of the sampling process:

o On page 5, line 12–31, you mention contacting all participants of CARE75+ who met inclusion/exclusion criteria.

o On page 5, line 40, you reference using theoretical sampling to support theory generation.

o Then, on page 7, it seems interviews were conducted with all contacted older people who consented.

3. Conclusion (p.20)

The conclusion currently places emphasis on the role of individual healthcare providers, but your findings also highlight systemic and ideological influences (e.g., normalization or trivialization of pain in older age). It would be important to reflect more critically on these broader factors. Otherwise, the conclusion risks inadvertently placing undue blame on individuals while overlooking institutional or policy-level responsibility. Could this framing trigger guilt among staff and let institutions off too lightly? Expanding the conclusion to acknowledge this would strengthen the paper's impact.

Please clarify how and when theoretical sampling occurred within this process. Specifically, how were participants selected at different stages, and how did those choices align with the principles of theoretical sampling?

Minor Revisions and Clarifications

• p. 4, l. 14:

"Consequently" may not be the best connector here. Consider using "On the other hand" or "In addition," depending on the intended logical flow.

• p. 6, l. 23:

If there was a specific cut-off value used for the Fried frailty criteria score, this should be explicitly reported.

• p. 6, l. 52:

The sentence "as were the second interview transcripts" is unclear in context. Please rephrase or clarify what is meant here.

Conclusion:

This manuscript is of high quality and provides important insights into the interplay between frailty, chronic pain, and healthcare organization. With the above minor revisions, it will be well positioned for publication.

**Reviewer**	**2**
**Name**	**Briggs, Andrew**
**Affiliation**	**Curtin University, School of Physiotherapy and Exercise Science**
**Date**	**10-Jul-2025**
**COI**	**None**

Thank you for the invitation to review this paper which reports on a primary qualitative study that explores the experience if older people living with pain and frailty and their care seeking decisions. The POPPY study and this nested POPPY-Q study are important in the context of the UN Decade of Healthy Ageing.

Title

• Add 'persistent' to the title to classify the pain cohort

Abstract

• Design: the design would be more correctly positioned as a cross-sectional study using qualitative methods. 'Qualitative' is a methodology, not a design. Similarly, this should be reflected in the Design section of the methods.

• It would be helpful to include in the Abstract some information about the level of frailty (as included in the Strengths and Limitations)

Background

• Para 1 describing the aetiology of chronic pain in older people is not really accurate. Chronic pain in older people may be secondary (eg from arthritis, or other condition) or can be primary(1). Both are important. One of the most common presentations is low back pain in older people. I refer the authors to the WHO Guideline for chronic primary low back pain for context on LBP in older people and refine this introduction so the classification and epidemiology narratives are more accurate(2).

• Para 2 – 65 years, rather than year

• Para 3 – it may be helpful to naïve readers to position pain and frailty and bi-directional

Methods

• The qualitative methods described are appropriate and well positioned, thankyou

• The PPI section is well described and in this context, your reporting would be strengthened by adding the GRIPP2 checklist (short form).(3)

• The Recruitment section blends recruitment and sampling together. My recommendation is to disaggregate this para into two: Sampling and then Recruitment, to strengthen and clarify the information. Related to this, please explain how participants were identified and invited from CARE75+ study and in temporal relation to when were details about pain medicines or diagnoses collected and interrogated; eg what is a 'routine CARE75+ assessment', and when does this happen? While the inclusion criteria are clear, there is a lack of detail here on the process to identify, recruit and enrol participants, which may introduce a sampling bias and perhaps collides with theoretic sampling. Figure 1 is helpful, but some more context in the Recruitment section would help. Please also provide the categories used for theoretic sampling as they relate to backgrounds, living circumstances and geographical locations.

• Screening: should this be NH or RM (not and)?

• Please consider the objectives against the focus of the 2 interviews as there is a slight disconnect between these. From the description of the interventions, the experiences with care-seeking seems to be a sub-point of interview #2, which is not how the objectives are described. Also the results and discussion do not refer to what service models could work – this part seems out of place to me.

• The sampling approach refers to theoretic sampling to support theory generation, yet the following sentences appear to refer to maximal heterogeneity sampling, rather than sampling to test/generate a theory. These are different concepts but appear conflated in the description provided. In theoretic sampling one generally targets sampling to explore and test an emerging theory. The description needs more context about how theoretic sampling was undertaken, particularly in reference to the CARE75+ database and how sampling and analysis were undertaken in real time to generate theory. I do wonder if what the authors actually did was a phenomenological study that explores lived experience where thematic map is created.

• The Data Analysis section I feel is lacking detail, especially for a grounded theory approach. Please revise this section to provide more detail about the explicit and sequential stages in the model (theory) development, in particular, the requirement for the constant comparative analysis. Typically, coding should be described in stages including open coding, axial coding and constant comparative analysis to ensure and describe how the emerging remains grounded within the data. Further, how did other members of the research team validate the coding framework and emergent theory?

• The Reflexivity statements is not really fit for purpose. This statement should focus on the positionality of the research team within the context of the work. Please revise this and also include the Rigour statement.(4-6)

Results

• Of the 102 invitations sent, what period of time were these sampled from with reference to the CARE75+ database? Specifically, what was the time difference between the data capture in the database and recruitment. Also, of the 102, how many declined and how many did not respond? Its not really clear how 102 selected from the database refers to theoretic sampling. If 26/102 agreed and were interviewed, how was theoretic or maximal heterogeneity sampling undertaken? The description reads more like a convenience sample that in the end was fortuitously heterogeneous.

• I recommend that Appendix be presented in the main MS as these data are essential to interpretation. A couple of issues here:

o I think you title should say "Data are frequencies…"

o Provide a description of the footer about decile rank, including reference, interpretation categories etc

• Table 1 should also include gender (or sex). Can you include information about the nominated pain conditions and standard minimum data on pain such as severity and impact(7)? It would also be helpful to colour code the frailty categories (like a heat map) so the heterogeneity can be easily assessed.

• Figure 2 outer categories are described as being inter-linked, yet the figure doesn't really show this. Perhaps consider a different design here, eg all part of an outer circle. The relationship between the outer and inner categories is not described or represented in the Figure. There appears to be an inter-relationship between the 2 inner categories, but this isn't really discussed.

• The authors need to provide more rationale as to why only 2 of the 6 categories are presented in this paper. The contextual relevance is provided, ie the link to the research program, but this potentially creates problems in analysis and interpretation as grounded theory. In many situations categories are not mutually exclusive (as directly mentioned by the authors) so there could be a reporting bias problem. Further, the analysis (in my opinion) cannot be positioned as grounded theory when the theory is not explained in its full form. It might be reasonable to drill down to 2 elements of the theory, but these cannot be presented in isolated to the other parts of the model in a grounded theory analysis. Is this actually a phenomenology study?

• For tables 2 and 3 to be stand-alone, it would be helpful to provide a narrative for each sub-category, so these could be interpreted against the quotes.

• Given the reported efforts for sample diversity, are there are signals about differences in experiences by age, gender, or other variable?

Discussion

• The Discussion is well positioned against the existing literature. However, I suggest that some structural considerations be made to optimize the flow. For example, Fig 2 depicts an inter-relationship between Acceptance and Support seeking decisions. As such, a para on each components of the model and then their interactions on a background of other factors, might be appropriate. In this way, the model structure is faithfully presented in the Discussion.

• The section on Frailty appears to be introducing new data that have not been included in the Results. I think this may not be appropriate here.

• Implications section is well positioned and important. Perhaps this could be framed as practice and policy implications. For practice, the results also suggest the need to consider pain care in older people who may not express pain as bothersome (eg case finding), that pain care needs to consider level of acceptance (or not) and care seeking attitudes, and ageist attitudes in community. From a policy perspective, integrated care for older people whether pain care is part of care for other NCDs may be relevant, such as the WHO model of healthy ageing and intrinsic capacity. Here the ICOPE model would serve well, with the most recent edition featuring the importance of pain assessment and management(8).

1. Nicholas M, Vlaeyen JWS, Rief W, Barke A, Aziz Q, Benoliel R, et al. The IASP classification of chronic pain for ICD-11: chronic primary pain. Pain. 2019;160(1):28-37.

2. World Health Organization. WHO guideline for non-surgical management of chronic primary low back pain in adults in primary and community care settings. Geneva: WHO; 2023.

3. Staniszewska S, Brett J, Simera I, Seers K, Mockford C, Goodlad S, et al. GRIPP2 reporting checklists: tools to improve reporting of patient and public involvement in research. BMJ (Clinical research ed. 2017;358:j3453.

4. Forero R, Nahidi S, De Costa J, Mohsin M, Fitzgerald G, Gibson N, et al. Application of four-dimension criteria to assess rigour of qualitative research in emergency medicine. BMC Health Serv Res. 2018;18(1):120.

5. Walsh R. The methods of reflexivity. The Humanistic Psychologist. 2003;31(4):51-66.

6. Ahmed SK. The pillars of trustworthiness in qualitative research. J Med Surg Public Health. 2024;2:100051.

7. Dworkin RH, Turk DC, Farrar JT, Haythornthwaite JA, Jensen MP, Katz NP, et al. Core outcome measures for chronic pain clinical trials: IMMPACT recommendations. Pain. 2005;113(1-2):9-19.

8. World Health Organization. Integrated care for older people (ICOPE): guidance for person-centred assessment and pathways in primary care, 2nd ed. Geneva: World Health Organization; 2024 2024-12-31. Report No.: 9789240103726 (electronic version)

9789240103733 (print version).

VERSION 1 - AUTHOR RESPONSE

REVIEWER 1

Many thanks for your very helpful suggestion and commends. We have compiled these in a table and provided responses.

Comments	Response
1. Abstract – Balance Between Methods and Results<br>The abstract currently devotes too much space to methodological details and too little to the study's key findings. This imbalance detracts from the paper's potential impact, especially for readers scanning abstracts to gauge relevance. Please revise the abstract to better reflect the central findings and their implications.	Thank you for highlighting this imbalance in the abstract. We have revised it to include more detail in the results and conclusions sections.
2. Clarify Sampling Strategy (POPPY-Q and CARE75+)<br>There appears to be some inconsistency in the description of the sampling process:<br>• On page 5, line 12–31, you mention contacting all participants of CARE75+ who met inclusion/exclusion criteria.<br>• On page 5, line 40, you reference using theoretical sampling to support theory generation.<br>• Then, on page 7, it seems interviews were conducted with all contacted older people who consented.	Thank you for highlighting this point. We have removed the following statement from page 5: *"Using theoretical sampling, we iteratively sampled and recruited participants to support theory generation."* We recognise that the term *theoretical sampling* was used in a broad sense and may have been misleading.<br><br>Figure 1 has been updated to provide greater clarity regarding the identification, sampling, and recruitment process. We have also added further detail to pages 6 and 7 to support transparency.<br><br>All individuals who provided consent were interviewed.
3. Conclusion (p.20)<br>The conclusion currently places emphasis on the role of individual healthcare providers, but your findings also highlight systemic and ideological influences (e.g., normalization or trivialization of pain in older age). It would be important to reflect more critically on these broader factors. Otherwise, the conclusion risks inadvertently placing undue blame on individuals while overlooking institutional or policy-level responsibility. Could this framing trigger guilt among staff and let institutions off too lightly? Expanding the conclusion to acknowledge this would strengthen the paper's impact.	Thank you, you raise an important point. We have added additional information at the end of the conclusion (Page 24).
4. Please clarify how and when theoretical sampling occurred within this process. Specifically, how were participants	Participants were invited over a number of mail-out rounds with later rounds focusing

selected at different stages, and how did those choices align with the principles of theoretical sampling?	on those with more moderate to severe frailty who were under-represented in the earlier mail outs. We have included this information in the manuscript (Page 6-7).<br><br>We have discussed the limitations of the theoretical approach within the 'Strengths and limitation' section of the discussion.
• p. 4, l. 14: "Consequently" may not be the best connector here. Consider using "On the other hand" or "In addition," depending on the intended logical flow.	Completed.
• p. 6, l. 23: If there was a specific cut-off value used for the Fried frailty criteria score, this should be explicitly reported.	The cut-points for the Fried frailty are reported on Page 6
• p. 6, l. 52: The sentence "as were the second interview transcripts" is unclear in context. Please rephrase or clarify what is meant here.	Amended on page 8.

REVIEWER 2

Many thanks for your very helpful suggestion and commends. We have compiled these in a table and provided responses.

Comments	Responses
Title<br>• Add 'persistent' to the title to classify the pain cohort	Thank you, the title has been amended.
Abstract<br>• Design: the design would be more correctly positioned as a cross-sectional study using qualitative methods. 'Qualitative' is a methodology, not a design. Similarly, this should be reflected in the Design section of the methods.	We have added the term *'qualitative methods'* in the design paragraph of the abstract and in the design section of the methods.<br><br>However, following a team discussion, we would prefer not to describe the study as a *cross-sectional study using qualitative methods*, as it is separate from the cohort from which we recruited participants.
• It would be helpful to include in the Abstract some information about the level of frailty (as included in the Strengths and Limitations)	This has been revised in the abstract.

Background<br>• Para 1 describing the aetiology of chronic pain in older people is not really accurate. Chronic pain in older people may be secondary (eg from arthritis, or other condition) or can be primary(1). Both are important. One of the most common presentations is low back pain in older people. I refer the authors to the WHO Guideline for chronic primary low back pain for context on LBP in older people and refine this introduction so the classification and epidemiology narratives are more accurate(2).	Thank you for this suggestion. We have incorporated primary and secondary pain into the background (introduction) section. We have referenced the NICE guidelines, which we hope is acceptable.
• Para 2 – 65 years, rather than year	We have made this change.
• Para 3 – it may be helpful to naïve readers to position pain and frailty and bi-directional	Thank you for this suggestion. We have given it careful consideration. However, we feel that the evidence supporting a direct link between frailty causing pain is not yet strong enough. As such, we would prefer not to include this.
Methods<br>• The qualitative methods described are appropriate and well positioned, thankyou	Thank you for this comment.
• The PPI section is well described and in this context, your reporting would be strengthened by adding the GRIPP2 checklist (short form).(3)	Thank you, we have completed the GRIPP2 checklist which has been added in supplementary data.
• The Recruitment section blends recruitment and sampling together. My recommendation is to disaggregate this para into two: Sampling and then Recruitment, to strengthen and clarify the information. Related to this, please explain how participants were identified and invited from CARE75+ study and in temporal relation to when were details about pain medicines or diagnoses collected and interrogated; eg what is a 'routine CARE75+ assessment', and when does this happen? While the inclusion criteria are clear, there is a lack of detail here on the process to identify, recruit and enrol participants, which may introduce a sampling bias and perhaps collides with theoretic sampling. Figure 1 is helpful, but some more context in the Recruitment section would help. Please also provide the categories used for theoretic sampling as they relate to backgrounds, living circumstances and geographical locations.	We have separated the recruitment and sampling as suggested and we have added further detail which we hope provides clarity. We now have three sections:<br>• Identification of potential POPPY-Q participants from the Community Ageing Research 75+ (CARE75+) study<br>• Sampling for POPPY-Q<br>• Recruitment to POPPY-Q<br><br>Additionally, we have included an updated diagram to explain the process (Figure 1).
• Screening: should this be NH or RM (not and)?	Completed.

• Please consider the objectives against the focus of the 2 interviews as there is a slight disconnect between these. From the description of the interventions, the experiences with care-seeking seems to be a sub-point of interview #2, which is not how the objectives are described.	The objectives and data collection sections have been amended to clarify the focus of each interview. The topic guide for interview 2 has been added to supplementary data (in addition to the topic guide for interview 1) to highlight the differences in focus.
Also the results and discussion do not refer to what service models could work – this part seems out of place to me.	Service models were not a feature within the study findings as very few people had any experience of specific services for their pain. However, that in itself is important information and we have reported this within the discussion (Page 21).
• The sampling approach refers to theoretic sampling to support theory generation, yet the following sentences appear to refer to maximal heterogeneity sampling, rather than sampling to test/generate a theory. These are different concepts but appear conflated in the description provided. In theoretic sampling one generally targets sampling to explore and test an emerging theory. The description needs more context about how theoretic sampling was undertaken, particularly in reference to the CARE75+ database and how sampling and analysis were undertaken in real time to generate theory. I do wonder if what the authors actually did was a phenomenological study that explores lived experience where thematic map is created.	Sampling was designed where feasible to support theory generation. However, we acknowledge the limitations of our approach and have reported them in the 'Strengths and limitations' section of the discussion.
• The Data Analysis section I feel is lacking detail, especially for a grounded theory approach. Please revise this section to provide more detail about the explicit and sequential stages in the model (theory) development, in particular, the requirement for the constant comparative analysis. Typically, coding should be described in stages including open coding, axial coding and constant comparative analysis to ensure and describe how the emerging remains grounded within the data. Further, how did other members of the research team validate the coding framework and emergent theory?	Thank you for your comment. We have redrafted this section providing more detail about the sequential stages of coding and model development including how this was validated by the research team.
• The Reflexivity statements is not really fit for purpose. This statement should focus on the positionality of the research team within the context of the work. Please revise this and also include the Rigour statement.(4-6)	Thank you for highlighting this issue. We have revised the section to focus on the positionality of the research team. Please see supplementary data for the COREQ checklist which provides an assessment of rigour.

Results<br>• Of the 102 invitations sent, what period of time were these sampled from with reference to the CARE75+ database? Specifically, what was the time difference between the data capture in the database and recruitment. Also, of the 102, how many declined and how many did not respond? Its not really clear how 102 selected from the database refers to theoretic sampling. If 26/102 agreed and were interviewed, how was theoretic or maximal heterogeneity sampling undertaken? The description reads more like a convenience sample that in the end was fortuitously heterogeneous.	Please see figure 2 which provides details of those consented, and those that could not be contacted or declined. This has enabled us to remove some of the narrative in that section. We hope this diagram is helpful.
• I recommend that Appendix be presented in the main MS as these data are essential to interpretation. A couple of issues here:<br>o I think you title should say "Data are frequencies…"<br>o Provide a description of the footer about decile rank, including reference, interpretation categories etc	We have moved the appendix data into the table and the data is no longer being reported as frequencies.<br><br>The information on IMD deciles has been presented within the text rather than incorporated into the table. This change has enabled us to include the information on sex and comorbidities. Some concerns were raised within the team regarding the inclusion of deciles alongside ethnicity, as this could potentially make some participants identifiable. As such, the IMD data are reported in the text.<br><br>An explanation of IMD deciles and an accompanying reference have been included.
• Table 1 should also include gender (or sex). Can you include information about the nominated pain conditions and standard minimum data on pain such as severity and impact(7)? It would also be helpful to colour code the frailty categories (like a heat map) so the heterogeneity can be easily assessed.	We have included a column for sex in Table 1. With respect to pain, detailed information of this nature was not available. Data on pain and pain severity obtained from the CARE75+ study were collected solely to facilitate the identification of individuals for study invitation. In some cases, identification was based on the presence of comorbidities (e.g., osteoarthritis) or the use of relevant medication (e.g., paracetamol). These data were therefore used to identify potential participants, with inclusion determined through discussion in accordance with the study protocol. Most participants were living with multiple long-term health conditions and experienced pain from several sources; consequently, a single, clearly defined source of pain was rarely

	identifiable. However, the number of comorbidities has been included, as this information was collected and is reported in Table 1.<br>Following discussion within the team, we decided not to include a heat map in the table.
• Figure 2 outer categories are described as being inter-linked, yet the figure doesn't really show this. Perhaps consider a different design here, eg all part of an outer circle. The relationship between the outer and inner categories is not described or represented in the Figure. There appears to be an inter-relationship between the 2 inner categories, but this isn't really discussed.	We have redesigned this. See Figure 3, to show the outer categories as contextual, background information. We hope that, together with the accompanying narrative in the manuscript, this approach provides greater clarity.
• The authors need to provide more rationale as to why only 2 of the 6 categories are presented in this paper. The contextual relevance is provided, ie the link to the research program, but this potentially creates problems in analysis and interpretation as grounded theory. In many situations categories are not mutually exclusive (as directly mentioned by the authors) so there could be a reporting bias problem. Further, the analysis (in my opinion) cannot be positioned as grounded theory when the theory is not explained in its full form. It might be reasonable to drill down to 2 elements of the theory, but these cannot be presented in isolated to the other parts of the model in a grounded theory analysis. Is this actually a phenomenology study?	Thank you for this comment, which generated discussion and reflection within the team, and has helped to clarify our decisions.<br><br>We did not feel it was useful to readers, nor to our development of a practical model for use in services, to elaborate on well-established areas of knowledge about elderly frail people and their relationship to healthcare, nor that of the impact of pain on everyday life, including in older adults and people with multiple comorbidities. Our findings added nothing to the existing extensive literature but were an essential part of our contextualising our interview, and our interviewees contextualising their experiences. However, we believe that readers of this journal will be sufficiently familiar with those to appreciate our focus on the two areas which we foreground, and which have not been reported before in this population. We recognise that this is a somewhat unusual approach to our material, but feel that it is justified, and that our text and figure now better relates all parts of the model in a way which we neglected to do in the previous version.
• For tables 2 and 3 to be stand-alone, it would be helpful to provide a narrative for each sub-category, so these could be interpreted against the quotes.	Thank you for your suggestion. We have added some narrative for each sub-category in tables 2 & 3.

• Given the reported efforts for sample diversity, are there signals about differences in experiences by age, gender, or other variable?	Thank you for this valuable suggestion. Unfortunately, the sample size in the present study was insufficient to permit robust subgroup analyses. We agree that the relationship between ethnicity and pain is an important area and warrants further investigation in this population.
Discussion<br>• The Discussion is well positioned against the existing literature. However, I suggest that some structural considerations be made to optimize the flow. For example, Fig 2 depicts an inter-relationship between Acceptance and Support seeking decisions. As such, a para on each components of the model and then their interactions on a background of other factors, might be appropriate. In this way, the model structure is faithfully presented in the Discussion.	Thank you for your comments. We have now added more detail about each of the components of the model and how acceptance and support seeking decisions are inter-related. See page 12
• The section on Frailty appears to be introducing new data that have not been included in the Results. I think this may not be appropriate here.	We have now reported frailty within the results. See page 10.
Implications section is well positioned and important. Perhaps this could be framed as practice and policy implications. For practice, the results also suggest the need to consider pain care in older people who may not express pain as bothersome (eg case finding), that pain care needs to consider level of acceptance (or not) and care seeking attitudes, and ageist attitudes in community. From a policy perspective, integrated care for older people whether pain care is part of care for other NCDs may be relevant, such as the WHO model of healthy ageing and intrinsic capacity. Here the ICOPE model would serve well, with the most recent edition featuring the importance of pain assessment and management(8).	Thank you for this suggestion. Please see updated 'Practice and Policy Implications' section on page 22-23.

VERSION 2 - REVIEW

**Reviewer**	**1**
**Name**	**Weber, Orest**
**Affiliation**	**Lausanne University Hospital, Psychiatric Liaison Service**
**Date**	**08-Sep-2025**
**COI**	

All the observations raised in my initial review have been adequately addressed. From my perspective, the manuscript is now suitable for publication.

**Reviewer**	**2**
**Name**	**Briggs, Andrew**
**Affiliation**	**Curtin University, School of Physiotherapy and Exercise Science**
**Date**	**09-Sep-2025**
**COI**	

Thank you for considering my suggestions. The paper has improved significantly in this revision, in my opinion.

I do have a few more suggestions to optimise accuracy

1. Contextualising chronic pain (Introduction). In my view, the first and third sentences of the para 1 collide. I suggest the authors re-frame this to explain that both chronic primary pain (not primary chronic pain) syndromes and chronic secondary pain syndromes are common in older people and may co-exist. Chronic primary pain describes a pain experience that cannot be better explained by an underlying pathology, consistent with ICD-11 classification. A better reference to use is Nicholas et al 2019(1). The NICE guideline has been criticised in the field.

2. Figure 1 is helpful, thank you. Should this read 'persistent pain'? Same for the first para on sampling – "living with persistent pain"?

3. Sampling: thank you for considering my comments about the sampling approach and how theoretic sampling was not faithfully applied in this sense. The authors have acknowledged, in the Discussion, that their approach was more purposive than targeted to theory generation. I agree with this and my recommendation here is to frame the sampling as such – ie say what it is. I think the purposive sampling to achieve maximum heterogeneity to develop a broad theory is reasonable, rather than trying to argue for theoretic sampling. I suggest re-frame the methods, Discussion, and Strengths and Limitations sections with this in mind.

4. The authors have not provided a rigour statement. The COREQ-32 is a reporting checklist, not a rigour statement. I recommend a discrete rigour statement be added, consistent with guidelines offered in my earlier comments(2, 3).

5. Fig 2 on flow is helpful. To optimise this, consider designing aligned to the STROBE standard. This would strengthen the Figure. The Figure provides critical data on selection and enrolment, however, the issue about the temporal relationship between data collection on the CARE75+ database and recruitment is not clear. Can the authors provide context

here? It is important to understand the potential time lag between selection variables and enrolment, and then data collection. I can see some information about this is presented in limitations, but best practice reporting would be also to include in the Methods as a specific attribute of sampling.

6. The authors have provided an explanation about why the central parts of the model presented in Fig 3 are the central parts of the results and why the other 4 themes are positioned as contextual and not reported in detail. I accept this rationale and understand the authors' decision. I have 2 suggestions in this regard:

a. Include a coding tree as a supplementary file so that you can show (for readers who are interested, such as those undertaking systematic reviews) the sub-categories and raw data for those 4 categories that are not described in detail (general health and wellbeing; pain and its impact; experience of accessing pain support and perception and experience of pain support and treatment).

b. The central themes "Acceptance of pain" and "Support seeking decisions" appear to be influenced (based on Fig 3) by pain impact and accessing pain support, based on a common green shading. However, the text on p.12 doesn't really explain these mechanisms. Rather, there is information about how the 4 contextual factors intersect, omitting the intersection between these contextual factors and the 2 central factors. Providing a mechanistic paragraph or interpretation about how pain impact and access pain support influence pain acceptance and support seeking decisions would close the loop on the model and better rationalise omitting detail from the 4 contextual categories.

References

1. Nicholas M, Vlaeyen JWS, Rief W, Barke A, Aziz Q, Benoliel R, et al. The IASP classification of chronic pain for ICD-11: chronic primary pain. Pain. 2019;160(1):28-37.

2. Forero R, Nahidi S, De Costa J, Mohsin M, Fitzgerald G, Gibson N, et al. Application of four-dimension criteria to assess rigour of qualitative research in emergency medicine. BMC Health Serv Res. 2018;18(1):120.

3. Ahmed SK. The pillars of trustworthiness in qualitative research. J Med Surg Public Health. 2024;2:100051.

VERSION 2 - AUTHOR RESPONSE

REVIEWER 1

Many thanks for deeming our manuscript ready for publication.

REVIEWER 2

Many thanks for your very helpful suggestion and commends. We have compiled these in a table and provided responses.

Comments	Responses
Contextualising chronic pain (Introduction). In my view, the first and third sentences of the para 1 collide. I suggest the authors re-frame this to explain that both chronic primary pain (not primary chronic pain) syndromes and chronic secondary pain syndromes are common in older people and may co-exist. Chronic primary pain describes a pain experience that cannot be better explained by an underlying pathology, consistent with ICD-11 classification. A better reference to use is Nicholas et al 2019(1). The NICE guideline has been criticised in the field.	We have updated the paragraph and included the proposed reference by Nicholas et al. See page 3.
Figure 1 is helpful, thank you. Should this read 'persistent pain'? Same for the first para on sampling – "living with persistent pain"?	We are glad you found figure 1. helpful.<br><br>We only reported 'pain' in Figure 1, not 'persistent pain' prior to consent, because when we received the contact list from the CARE75+ study team, these were potentially eligible participants. We did not have any information about the type of pain or how long they had experienced pain for. At that stage, they had not yet consented to take part in POPPY, so only very limited information was available. Study eligibility was discussed during the first conversation.<br><br>Additionally, as reported in the sampling section: "*The CARE75+ project manager screened the CARE75+ database to identify those who had agreed to be contacted about other studies, were living with frailty, and had reported pain or pain impact…*" Again, at this stage, the list gave us no indication of whether the person had persistent pain, as we were being provided with a list of potentially eligible older adults - with eligibility still to be determined.
Sampling: thank you for considering my comments about the sampling approach and how theoretic sampling was not faithfully applied in this sense. The authors have acknowledged, in the Discussion, that their approach was more purposive than targeted to theory generation. I agree with this and my recommendation here is to frame the sampling as such – ie say what it is. I think the purposive	The authors agree with this comment, and we have reframed the sampling approach as purposive rather than theoretical, with an acknowledgment that this is a minor deviation from the study protocol, in the methods sections on page 7. We have removed this sentence from the strengths and limitations sections on page 2 and page 22 as we do not feel it constitutes a limitation.

Commented [AW1]: I've suggested taking it out of the limitations p22 as purposive sampling is not a limitation.

sampling to achieve maximum heterogeneity to develop a broad theory is reasonable, rather than trying to argue for theoretic sampling. I suggest re-frame the methods, Discussion, and Strengths and Limitations sections with this in mind.	
The authors have not provided a rigour statement. The COREQ-32 is a reporting checklist, not a rigour statement. I recommend a discrete rigour statement be added, consistent with guidelines offered in my earlier comments(2, 3).	The following rigour statement has now been included at the end of the methods section on page 10.<br><br>*To improve rigour and trustworthiness, reflexivity was embedded throughout the research process. NH and RM engaged in ongoing critical reflection by maintaining detailed reflexive memos to document potential biases and assumptions alongside regular analytical discussions with colleagues. We did not check the content of the transcripts with POPPY-Q participants as we considered this task to be too burdensome for an older frail population who had already undertaken two interviews for this study. There was, however, substantial PPI involvement in the analytical process which provided an external perspective, helping to validate the findings. These strategies collectively enhanced the credibility, dependability, confirmability and transferability of the study.*
Fig 2 on flow is helpful. To optimise this, consider designing aligned to the STROBE standard. This would strengthen the Figure. The Figure provides critical data on selection and enrolment, however, the issue about the temporal relationship between data collection on the CARE75+ database and recruitment is not clear. Can the authors provide context here? It is important to understand the potential time lag between selection variables and enrolment, and then data collection. I can see some information about this is presented in limitations, but best practice reporting would be also to include in the Methods as a specific attribute of sampling.	We are glad you found figure 2 helpful. Following discussion, we have decided to retain the diagram in its current format. We think both figures 1 and 2 provide the necessary information clearly.<br><br>However, to address the issue of the temporal relationship, we added the following information on page 6.<br><br>*The above data represented the most recent information available from the CARE75+ dataset and varied for each potential participant. CARE75+ assessments were conducted between 3 months and 2 years prior to the list being shared with the POPPY-Q team. In some cases, participants had*

	already completed the CARE75+ study but had consented to be approached about other studies.<br><br>We hope this helps address this issue. Please note the POPPY-Q team were not provided with the specific dates of the assessments for the CARE75+ data. The list of contacts was shared with limited information and as noted by the reviewer, the potential time lag has been acknowledged in the limitations.
The authors have provided an explanation about why the central parts of the model presented in Fig 3 are the central parts of the results and why the other 4 themes are positioned as contextual and not reported in detail. I accept this rationale and understand the authors' decision. I have 2 suggestions in this regard:<br>a. Include a coding tree as a supplementary file so that you can show (for readers who are interested, such as those undertaking systematic reviews) the sub-categories and raw data for those 4 categories that are not described in detail (general health and wellbeing; pain and its impact; experience of accessing pain support and perception and experience of pain support and treatment).<br>b. The central themes "Acceptance of pain" and "Support seeking decisions" appear to be influenced (based on Fig 3) by pain impact and accessing pain support, based on a common green shading. However, the text on p.12 doesn't really explain these mechanisms. Rather, there is information about how the 4 contextual factors intersect, omitting the intersection between these contextual factors and the 2 central factors. Providing a mechanistic paragraph or interpretation about how pain impact and access pain support influence pain acceptance and support seeking decisions would close the loop on the model and better rationalise omitting detail from the 4 contextual categories.	Thank you for your feedback.<br><br>a) We have included a supplementary file that provides further detail about the contextual categories and sub-categories underpinning the conceptual model. This supplementary material is intended for readers who may wish to explore the model in more depth and better understand how the key domains were developed. I have referred to this supplementary file on page 13.<br><br>b) We have added the following paragraph to highlight how pain impact and access to pain support can influence pain acceptance and support-seeking decisions on page 12.<br><br>*These two inner categories more directly tied to participants' daily challenges of living with pain and their experiences of accessing support and have a stronger influence on attitudes towards pain and their decisions to seek support for pain.*

VERSION 3 - REVIEW

**Reviewer**	**2**
**Name**	**Briggs, Andrew**
**Affiliation**	**Curtin University, School of Physiotherapy and Exercise Science**
**Date**	**23-Sep-2025**
**COI**	

Thank you for considering my comments. I am satisfied the paper is methodologically sound and reported in alignment with contemporary standards.
